# Supplementary material for: Real-time, automated, standardized, and transparent analysis of microfluidic nanoparticle data with RPSPASS
Source: bioRxiv. 2026 Apr 1:2026.03.30.715405. Preprint. [Version 1] doi: 10.64898/2026.03.30.715405 (PMC13317600; doi:10.64898/2026.03.30.715405)
Supplement: Supplement 1 [file NIHPP2026.03.30.715405v1-supplement-1.pdf]

bioRxiv preprint doi: https://doi.org/10.64898/2026.03.30.715405; this version posted April 1, 2026. The copyright holder for this preprint (which was not certified by peer review) is the author/funder, who has granted bioRxiv a license to display the preprint in perpetuity. It is made available under a CC-BY 4.0 International license.

Supplemental Figures

Real-time, automated, standardized, and transparent analysis of microfluidic nanoparticle data with RPS$_{PASS}$

Michelle L. Pleet[1], Sean Cook[2], Bryce Killingsworth[2], Tim Traynor[2], Dove-Anna Johnson[2], Emily H. Stack[1], Verity Ford[3], Cláudio Pinheiro[4,5], Jessie E Arce[6], Jason Savage[2], Matthew Roth[6], Aleksandar Milosavljevic[6], Ionita Ghiran[7], An Hendrix[4,5], Steven Jacobson[1], Joshua A. Welsh[2†], Jennifer C. Jones[2†*]

Supplemental Information 1. The complete MIFlowCyt-EV report can be accessed at: https://figshare.com/s/bcd06197af644df99430

bioRxiv preprint doi: https://doi.org/10.64898/2026.03.30.715405; this version posted April 1, 2026. The copyright holder for this preprint (which was not certified by peer review) is the author/funder, who has granted bioRxiv a license to display the preprint in perpetuity. It is made available under a CC-BY 4.0 International license.

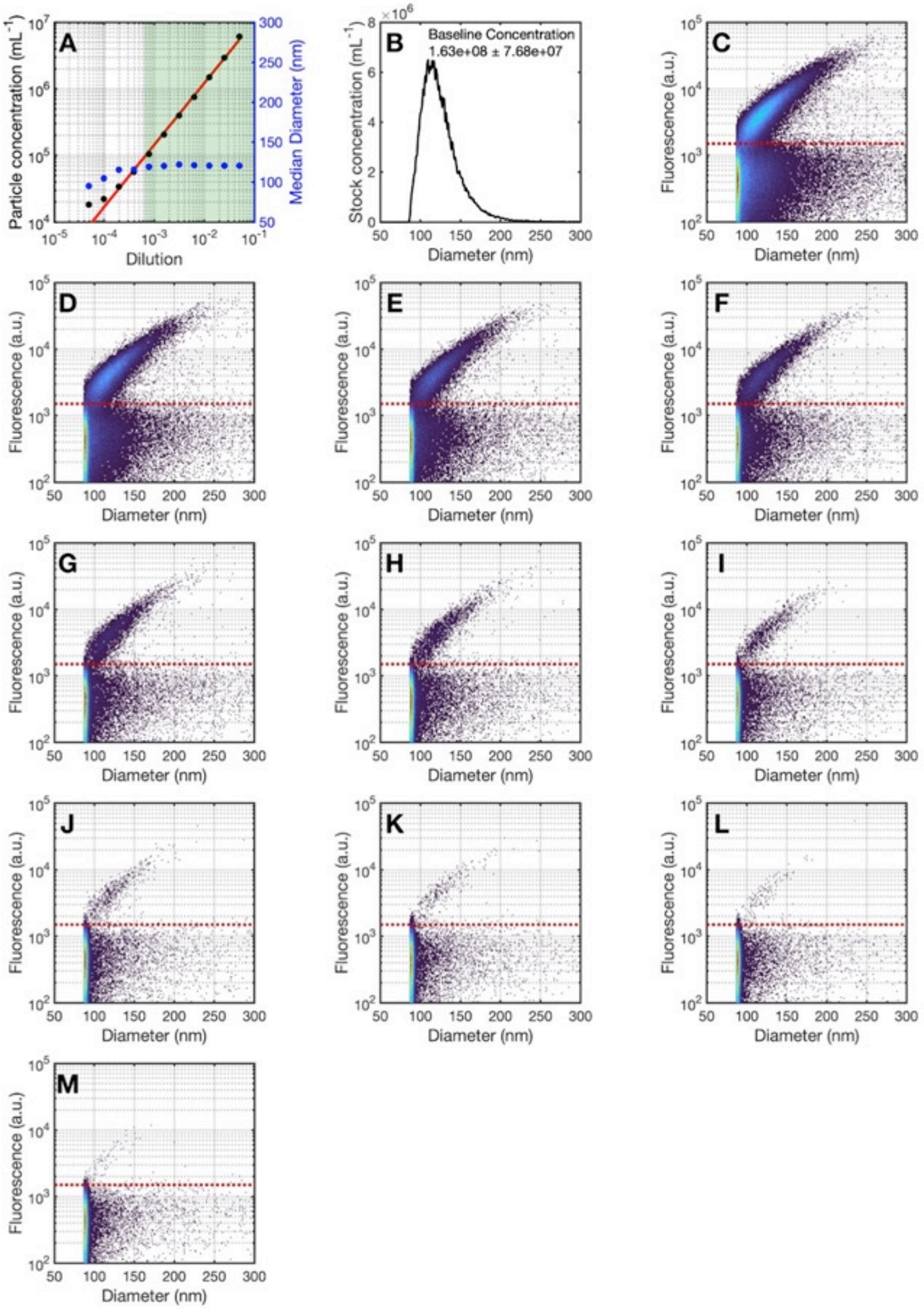

Supplemental Figure 1: Flow cytometry quality control data for rEV acquisition. A) serial dilution of rEVs with concentration vs. dilution factor (black dots, red regression line) and median light scatter derived diameter (blue dots). Green shaded area shows the most reliable concentration dynamic range of detection for rEVs. **B**) diameter distribution of rEVs using light scatter calibration with the detected concentration. **C-M**) show raw data plots from low dilution factor to high dilution factor.

bioRxiv preprint doi: https://doi.org/10.64898/2026.03.30.715405; this version posted April 1, 2026. The copyright holder for this preprint (which was not certified by peer review) is the author/funder, who has granted bioRxiv a license to display the preprint in perpetuity. It is made available under a CC-BY 4.0 International license.

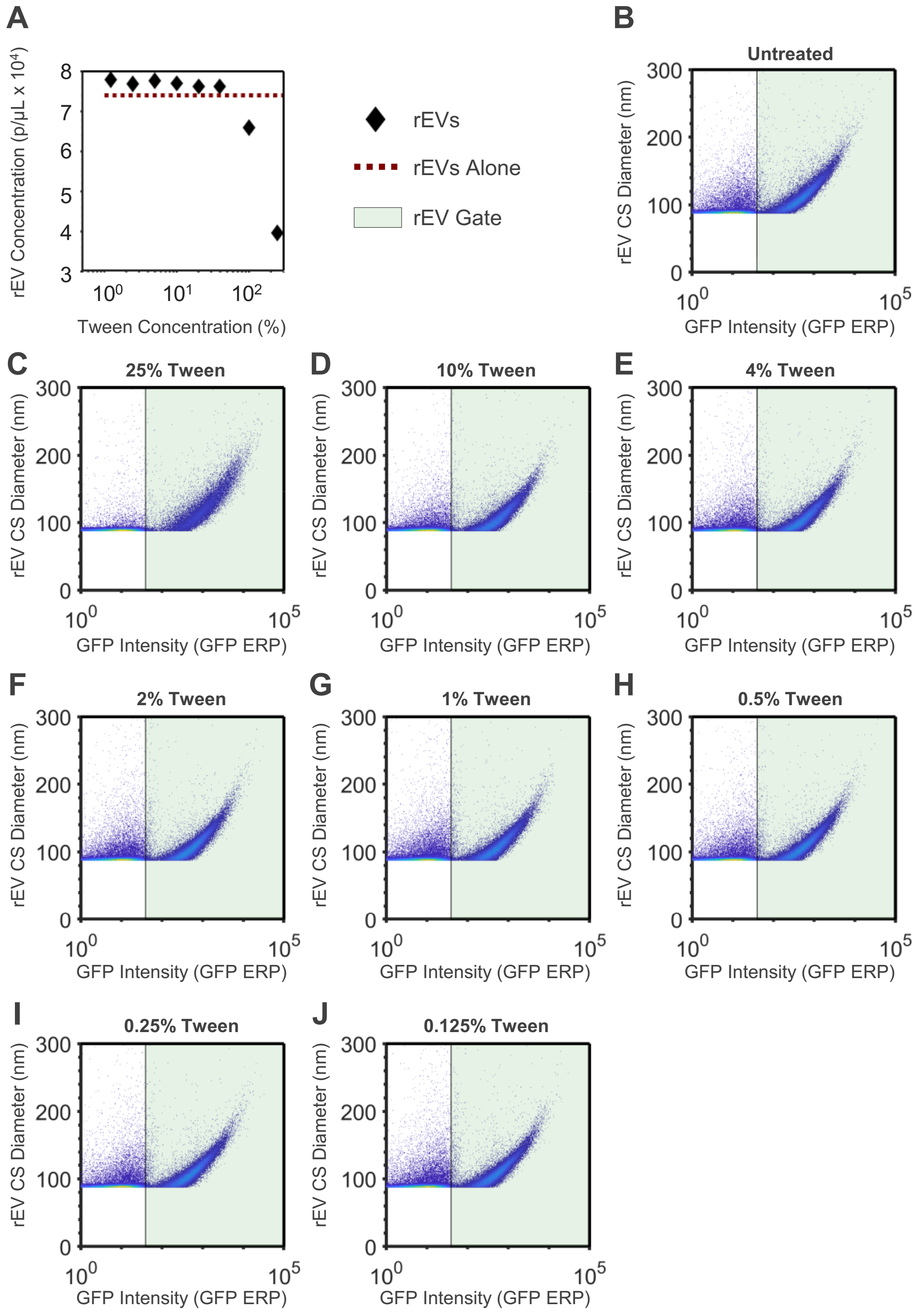

Supplemental Figure 2: Tween titration control data for rEV acquisition. A) Summary flow cytometry data of rEVs (black dots) with an increasing titration of Tween 20. rEVs alone with no Tween 20 treatment are shown (red dotted line). **B-J**) Raw data plots of each rEV +/- Tween 20 treatment flow cytometry acquisitions. Green shaded areas show the selected rEV gate.
